# Risks and regulation of rubber scattershot in Switzerland: a narrative review

**DOI:** 10.1038/s41433-024-03215-w

**Published:** 2024-07-08

**Authors:** Anna Fierz

**Affiliations:** Ophthalmologist in private practice, Kalkbreitestr. 8, 8003 Zürich, Switzerland

**Keywords:** Risk factors, Physiology

## Abstract

Multiple kinetic impact projectiles (KIPs) are responsible for most eye injuries by crowd-control weapons. This review aims to outline an underreported, ongoing series of eye injuries by rubber scattershot in Switzerland, and to relate current knowledge about thresholds for lasting visual loss to the energy limits set on KIPs in crowd control, by way of a retrospective compilation of cases from publicly available records and a review of the pertinent literature. Scattershot can cause irreversible visual loss. Since 1980, there have been 36 known cases of eye injuries by rubber pellets in Switzerland. In 2023, the incidence was higher here than at the peak of protests in France. KIPs were originally cleared for use in crowd control at kinetic and area-normalised energies assumed to lie below the threshold for ocular penetration. However, closed globe injuries suffice to cause permanent visual loss. Lower energy thresholds for lasting damage have been confirmed by the newer literature on paintballs, airbags, air guns and toys. These values may differ in vivo versus in vitro, and in humans versus in animals. There is no clear consensus on how best to predict loss of vision. Underreporting the risks of crowd-control weapons may contribute to their prolonged and increasingly liberal use. Regulations should consider what is known on energy thresholds for permanent visual loss. It is critical for ophthalmologists to be involved in the evaluation and monitoring of eye injuries caused by projectiles, including KIPs and toys. An interdisciplinary approach could help to elucidate damage thresholds.

## Abstract (zh)

多重动能冲击弹(KIPs)造成大多数人群控制武器眼外伤的原因。

本文旨在通过对公开、记录的病例进行追溯和相关文献的综述, 概述在瑞士因橡胶散弹造成的一系列未被报道的、正在发生的眼外伤, 将当前关于永久性视力丧失阈值的知识与人群控制中对KIPs设定的能量限制联系起来。

散射体可导致不可逆性视力损伤。自1980年以来, 瑞士已知有36例因橡胶颗粒造成的眼外伤病例。2023年, 瑞士的发病率较法国抗议活动高峰时期更高。KIP最初批准用于人群控制, 其动能和面积归一化能量被认为低于眼部穿透阈值。然而, 闭合性眼球损伤足以导致永久性视力丧失。关于彩弹、安全气囊、气枪和玩具的最新文献证实, 造成持久损伤的能量阈值较低。这些值在体内和体外可能不同, 在人和动物中也可能不同。对于如何最好地预测视力丧失, 目前还未达成明确共识。

低估人群控制武器的风险可能会导致其长期和日益广泛的滥用。法规应考虑关于永久性视力丧失能量阈值的已知情况。眼科医生参与评估和监测投射物, 包括KIPs和玩具造成的眼外伤至关重要。跨学科有助于阐明损害阈值。

## Introduction

Less-lethal weapons are in use around the world for crowd control. Their effects have been under scrutiny for years [1–3]. Reports on ocular injuries from kinetic impact projectiles (KIPs) have recently come from countries as diverse as India, the United States, Chile and France. Others have been using them for decades, like South Africa or Israel, while the United Kingdom has restricted their use.

KIPs come in many shapes and sizes, including multiple projectiles fired simultaneously from the same launcher, also known as scattershot. An extensive review of death and disability from KIPs [4] included a small series of eye injuries from rubber scattershot from Zürich in 2000/01 [5]. In Switzerland, such injuries first occurred in 1980, and they still happen sporadically. However, there are no other Swiss series of ocular trauma from KIPs in the medical literature, no reporting channels and no statistics. Trust in state authorities is traditionally high in our country [6], and the use of KIPs is widely regarded as normal here.

Injured patients often meet with disbelief. Several cases have been closed before making it to court. Prosecutors regularly suggest the damage could have been done by a stone, a fist, an elbow, or even that it could be self-inflicted. A prosecutor recently asserted that rubber shot causes no more than bruises [7] – after an unauthorised demonstration where 1500 scattershot projectiles were fired into a non-violent crowd in 80 seconds [8, 9]. These events raise the question whether criminal courts - and perhaps also decision-makers and the public - underestimate the risk of permanent or severe injury from KIPs.

## Methods

The roots of this work were countless conversations with colleagues over several years. Over time, these broadened to include witnesses to the Swiss youth unrests in the 80 s, journalists and legal professionals, as well as patients and their families, who started contacting me after my local publications on the subject in 2023. Advice and input not qualifying for authorship was widely sought from experts. Most of them did not wish to be acknowledged due to the controversial nature of the subject. From 2021 through May 2024, I compiled publicly available records from Zürich’s university libraries including those of the Swiss Federal Institute of Technology (for technical papers) and the Sozialarchiv (for historical ones about the 1980s), and from Swiss and international media. The pertinent medical literature was accessed via Medline, following up as many of the quoted sources as possible. (One paper was provided directly by the Association of Firearms and Tool Mark Examiners.)

The main literature search used Medline and combinations of the following terms:

Ocular injury OR eye injury OR ocular trauma AND either:

- Kinetic impact projectiles OR less-lethal weapons OR non-lethal weapons OR crowd control OR paintballs OR non-powder firearms OR air guns OR foam dart guns OR airbags
- Energy OR kinetic energy OR area-normalised energy OR energy threshold
- Post-mortem OR in vivo OR in vitro OR animal models OR porcine eyes OR pig eyes

Some papers not referenced in Medline were quoted in other articles among the references and retrieved via the university library.

### Approximate number of eye injuries by kinetic impact projectiles in Switzerland

During Zürich’s youth unrests in the 1980s, there were at least four eye injuries from rubber scattershot from a short distance that were severe enough to lead to enucleation or profound, permanent visual loss. The ensuing political discussion was apparently resolved by introducing a minimum range of 20 m. At the time, a practising ophthalmologist and an association of concerned parents attempted to warn authorities and the public. Their efforts found little resonance in Switzerland but probably contributed to Germany’s decision not to introduce similar ammunition. Ever since, the issue has been deemed “controversial” and “political” even among local ophthalmologists. There is reason to believe it is underreported, particularly in the early years. Some insurances initially refused coverage, and many employers disapproved of the young protesters, so patients sometimes invented cover stories [10, 11]. Even today, lawyers often advise injured subjects against bringing charges if they cannot prove they were uninvolved in riots, which appears to be the rule [personal communication from patients].

In 2021, our attempts to gather data from Swiss clinics failed [10]. Two years later, a compilation of publicly available records found thirty documented eye injuries from 1980 to early May 2023, eleven resulting in blindness [11, 12]. As in previously published case series of less-lethal projectile injuries [5, 13–29], the typical patient is a young male. The pattern is similar to paintball injuries. Profound visual loss is possible from the allegedly safe minimum distance, and extensive globe rupture at closer range [5, 11].

After this compilation, two additional injuries from 2021 to 2022 surfaced [30, 31], personal communication from patients or relatives] and four new ones occurred until November 2023, raising the known total to 36 [32, 33], personal communications from Dr. Martin Schmid and from another patient]. The incidence here in 2023 was higher than in France at the peak of the gilets jaunes protests 2018, when 25 cases were reported in a year [26].

So far, no Swiss case is publicly known of worst-case scenarios familiar with faster or heavier KIPs, such as brain injury [18, 21, 34–36] or death [4, 13, 14, 19, 20, 23, 37]. Other significant injuries appear to be rare. A ruptured testicle was reported in the press from a larger single projectile, not from scattering pellets [8]. Conversely, no eye injury from a single projectile has come to public attention. This would fit the previously described pattern of multiple KIPs accounting for 96% of eye injuries by KIPs reported in the international medical literature since 2016, almost all of which occurred in India and Chile [2].

### Types of kinetic impact projectiles used by law enforcement agencies in Switzerland and elsewhere

Switzerland introduced rubber bullets for crowd control in the late 1970s for use with a multi-purpose launcher (Mehrzweckwerfer, MZW) derived from a regular Swiss Army rifle, capable of firing packages of 35 hexagonal rubber prisms (Saltech, Switzerland). The ballistics of the projectiles have been detailed [38]. Where available, the technical details of KIPs currently in use in Switzerland are given in Table 1.

**Table 1:** Kinetic impact projectiles (KIPs) used by Swiss law enforcement agencies.

Type	Size (mm)	Weight (g)	Muzzle velocity (m/s)	Minimum distance (m)	Kinetic energy at MD (J)	Area-normalised energy at MD (J/m^2^)	Number of KIPs	Dispersion area at distance given (m)
MZW rubber prisms (Saltech)	Length 26-27, hexagon sides 10 [41, own measurement]	10 [38]	70 [38]	20	10.2 [38]	39 000 [38]	35	about 4×4 at MD [8]
GL-06 rubber shot (B&T)	Diameter 15 [41]	2.6 [41]	88 [41]	5 [42]	about 8 [41]	about 45 000	28 [41]	about 2.7×3.3 (at 20 m) [41]
GL-06 “Rubber Shot Hexagonal” (Saltech)	Length 18, hexagon sides smaller than in MZW prisms (Fig. 1)	8.7 [42]	?	10 [42]	?	?	28 [42]	allegedly smaller than for MZW rubber prisms [43]
GL-06 SIR (B&T)	40×99 [40]	77 [40]	85 [40]	5 [42]	about 100-110 [50]	about 83 000	1	-
Cougar-56 (Alsetex)	?	?	?	15 [44]	?	?	20 [109]	allegedly about 1×1 (at MD) [44]

*MZW* Mehrzweckwerfer, *MD* minimum distance, *SIR* “Safe Impact Round”.

In the 2000s, a single projectile named Flash-Ball^TM^ (Verney-Carron, France) was used by special forces in Lausanne [39].

Since the 2000s, some cantons (Swiss provinces) have introduced the GL-06 launcher (B&T, Switzerland) with several types of ammunition, including single projectiles (euphemistically named “Safe Impact Rounds”) and scattering pellets or “rubber shot” [40]. Different types of rubber shot are in use, mainly the manufacturer’s own spherical pellets [41]. One canton uses smaller hexagonal rubber prisms (Saltech) [42]. The kinetic energy of this smaller type of hexagonal rubber shot is not publicly known. Its area of dispersion is allegedly smaller, making it more precise but raising concern about multiple injuries among experts [43].

In 2024, the canton (Swiss province) of Vaud introduced the Cougar-56 (Alsetex, France), a 56 mm launcher, again with rubber scattershot. A spokesman for the police mentioned an area of dispersion of about 1×1 m at the minimum distance of 15 m without further details [44].

**Fig. 1:**
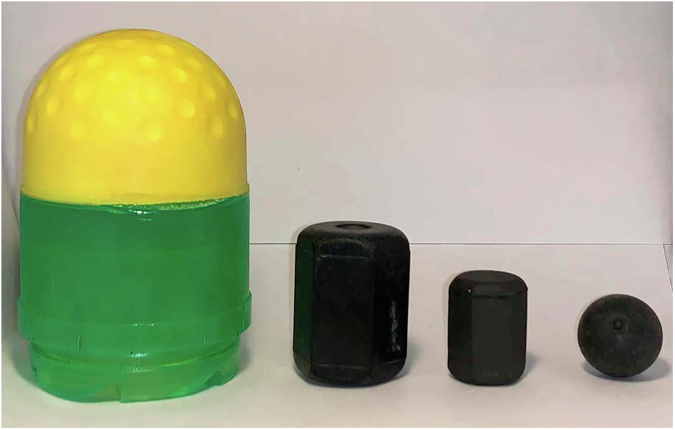
KIPs used in Switzerland in 2022. From left: Safe Impact Round (B&T), hexagonal rubber prisms for the MZW and the GL-06 (Saltech), spherical rubber shot for the GL-06 (B&T) [42] (reprinted with permission from republik.ch).

In recent cases, the projectile type is sometimes unknown. However, several of the latest injuries occurred in regions using the GL-06 and either B&T’s own rubber shot or Saltech’s for the GL-06. Except for the Flash-Ball, all these projectiles remain in use (Fig. 1).

Compared to KIPs from other countries, most of the Swiss projectiles are smaller and lighter. However, most other countries do not use multiple projectiles. In France, the GL-06 is called LBD-40 (for lanceur à balles de défense). The French only employ it with single projectiles. France is the only other country in Western Europe known to use multiple KIPs regularly, namely stun grenades that explode dispersing many KIPs. They should be deployed by hand by rolling them along the ground but have occasionally been thrown, for example in the case of Theron versus France [45]; personal communication from Neil Corney, Omega Research Foundation].

Some earlier KIPs were much larger: The original rubber bullets used by the British army in Northern Ireland were 15 cm long and weighed 135–140 g [13]; they were replaced in the mid-1970s by plastic bullets 10 cm in length and 3.7 cm in diameter. In total, 17 people were killed by rubber and plastic bullets in Northern Ireland. In the mid-2000s, the attenuating energy projectiles (AEPs) were introduced, similar in size to plastic bullets. AEPs are made of polyurethane with a crumple zone, i.e. an air gap in the nose [46]. They are carried in all Armed Response Vehicles in mainland UK, to provide a less lethal alternative to firearms, and regularly carried in Northern Ireland during policing of protests, particularly in the “marching season” [personal communication from Neil Corney, Omega Research Foundation]. Although rarely used, they have potential to cause serious injuries [46].

The same plastic bullets deployed in the UK from the mid-70s were later also used in South Africa [16] and in the early years of the Palestinian Intifada [18]. In Israel, they were superseded by other types, spherical or cylindrical, around 18–20 mm in diameter and length, some with a metal core [19, 21] and including multiple projectiles [20]. In recent years, there has been a rapid and often intransparent proliferation of different KIPs. Their evolution in the United States alone takes in-depth reporting to describe, from “stun guns” in 1970 to bean bag rounds and pepper balls [47]. Scattershot has been used in the USA and also in Chile, where it had a metal core and resulted in at least one well-documented case of bilateral blindness [48]. India uses birdshot (scattering metal pellets) in Kashmir [29]. The international market for less-lethal weapons continues to grow [2]. Protesting or even reporting their effects can be dangerous in countries that are not free, for example in Iran, which appears to use scattershot too, but details are unclear [49]. What we know is probably the tip of the iceberg.

### Energy thresholds for visual loss: the basis for regulations in Switzerland

**Fig. 2:**
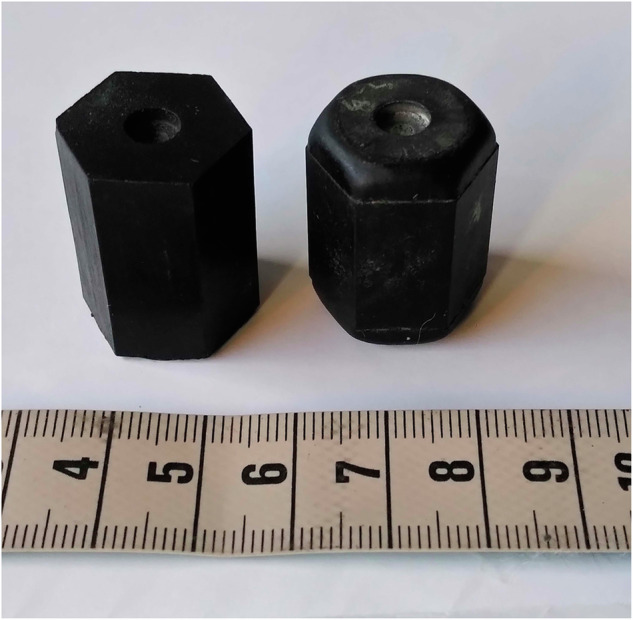
Older and newer rubber prisms for the MZW with sharp versus rounded corners (Saltech), scale: centimetres. (photo by the author).

When rubber bullets were introduced in Switzerland, authorities assumed thresholds for lasting eye damage from scattershot. Internal documents set limits for area-normalised impact energy: 25 000 J/m^2^ for “irreversible superficial eye damage, e.g. corneal abrasions”, and 60 000 J/m^2^ as the threshold for penetration [50]. They also set limits for kinetic energy: 10 J (the kinetic energy of the MZW rubber prisms at 20 m) as the minimum for “irreversible eye damage” [50]. The source for area-normalised impact energy is a textbook [51] referencing papers from the 1960s that determined threshold velocities for the penetration of rabbit eyes post-mortem [52, 53]. From these, the textbook’s editors calculated a threshold of 60 000 J/m^2^ for BB spheres. The numbers for cube-shaped projectiles of identical weights were significantly lower [51]. The Swiss prisms used to have sharp corners; they were rounded in the 2000s (Fig. 2). The same textbook gives the area-normalised impact energy for the MZW prisms as 39 000 J/m^2^ from 20 m when they hit the eye frontally with the hexagonal area first. Impact areas are customarily equated with the average area of the projectile’s shadow in the direction of the shot [51]. This makes sense for high-energy projectiles that penetrate rapidly. The question remains whether area-normalised energy is conceptually reliable for low energy projectiles, especially if they do not have a uniform profile but are polygonal [54, 55].

By implication, any harm done at lower energies was expected to be reversible. Although these figures probably reflect what was known in the 1970s, it seems safe to assume no ophthalmologists were consulted, as they would have known that penetration is not necessary to cause irreversible visual loss. Moreover, the newer literature on eye injuries by airbags, paintballs, air guns and toys was never considered. These assumptions therefore require correction.

### Energy thresholds for visual loss: what we know

Due to methodological challenges, there is no clear consensus as to what parameters best predict the risk of lasting damage from projectiles. It is usually impossible for clinicians to quantify the forces at work. To this end, researchers have experimented with various outcomes, from corneal abrasions to globe rupture, often looking for visible pathology in experimentally impacted animal eyes. In vitro, higher energy thresholds have been documented in porcine eyes than in human ones for iridodialysis [56] and for globe rupture [57]. Although some hold that “it is not possible to test post-mortem eyes for injury pathologies such as lens and retinal damage” due to their rapid degradation [57], others have found roughly reproducible patterns of injury with increasing amounts of impact energy in such tests [58, 59]. Small, fast projectiles cause more harm than larger, slower ones at similar levels of kinetic energy [57, 58, 60, 61]. Post-mortem findings were used to develop models for globe rupture [57, 60, 61] and later also numerical models presumed capable of reproducing observable patterns [62].

Investigators have looked at many parameters including mass, velocity, force, kinetic energy, momentum, area-normalised impact energy, rupture stress and intraocular pressure [63]. A review found area-normalised energy to be a better predictor of injury than kinetic energy [64]. This conclusion is probably more reliable for globe rupture than for closed globe injuries. For retinal detachment, the predictive value of kinetic energy was comparable to that of area-normalised energy [64]. Kinetic energy was still better than velocity or mass alone [57, 64] or diameter [57]. Previously, kinetic energy was shown to be a better predictor of injury than momentum [58] yet momentum has resurfaced in recent studies [55]. Stress and pressure are interesting research areas but of limited practical use. Intraocular pressure appears to correlate better with kinetic energy than with area-normalised energy [65]. Therefore, total kinetic energy may play a bigger role in closed globe injury than in open globe injury.

There is a wealth of data. A lot of the research comes from outside ophthalmology, for example from biomechanics experts investigating airbag injuries [57–62, 64, 65], or from forensic specialists [55, 66, 67]. Clinical observations include injuries from paintballs, air guns and even toys. Research has been done in enucleated human and animal eyes, mostly from pigs [55–59, 65–69], but also in live animals, particularly for retinal lesions difficult or impossible to produce post-mortem: for example, in rhesus monkeys [70], owl monkeys [71], pigs [72–75], rabbits and rats [76, 77].

### Clinical experience

In most cases, the condition of the retina (more precisely, the viability of the macula) determines the outcome of an injury [78]. Commotio retinae can lead to lasting visual impairment when it occurs in the macular region in about 26% [79] to 40% [80] of cases. The threshold for lasting damage is unclear [79]. Commotio retinae may occur without anterior segment pathology both as a coup and as a contrecoup lesion, albeit rarely [80]. The damage depends on the force with which the object hits the eye, and small high-speed objects do the greatest damage [80]. Other vision-threatening sequelae of ocular trauma include glaucoma, which can become manifest much later, and optic neuropathy. Like retinal damage, they are hard to model experimentally. While closed-globe injuries have a better prognosis than open-globe injuries on average, globe rupture or penetration is not a prerequisite for profound and permanent visual loss.

Thresholds for globe rupture appear to be lower in older or more myopic persons and after radial keratotomy [63]. Children and adolescents are overrepresented in many series of trauma and fatalities from KIPs and air guns, probably not only due to risk-taking behaviour but also to their more fragile anatomy [13–15, 17–21, 25, 81–84].

Paintballs are well known to cause devastating eye injuries including globe ruptures [85–89], but they come in many sizes and the kinetic energy of the offending projectiles is usually not mentioned in case series. Their weight is given as 3–3.5 g by several sources, either for the most common 0.68 inch = 17 mm caliber [87, 90] or for diameters of 14–17 mm [89]. At a muzzle velocity of 70 m/s, this would result in a muzzle energy around 8 J. Apparently paintball markers (guns) are capable of higher speeds, but these are limited in supervised settings. In paintball arenas, fewer injuries occurred after eye protection became mandatory [86]. In Germany, paintball markers with a muzzle energy above 7.5 J require a weapons license.

Airsoft guns can cause angle recessions and choroidal ruptures at area-normalised impact energies of 10 000 J/m^2^ [91]. Air guns have repeatedly caused fatalities [81–84, 92] despite low kinetic energies, leading to the 2015 Air Weapons and Licensing (Scotland) Act: guns with a muzzle energy over 1 J are no longer freely available in Scotland, while England and Wales retain older laws with limits of 16 J for air guns and 8 J for air pistols.

A comprehensive epidemiological investigation of pediatric non-powder firearms injuries over 26 years in the USA found a marked increase in eye injuries despite a decline in total injuries [93]. Recent evidence shows that even foam dart (Nerf) guns marketed as toys can cause not only traumatic hyphaemas [94] but also angle recession, vitreous hemorrhage, commotio retinae and severe retinal photoreceptor damage [95].

### Post-mortem studies and in vitro animal models

In vitro, globe rupture in human eyes occurs with BB guns starting at 1.36 J but not until around 70–80 J with baseballs [60]. Kennedy et al calculated a 50% risk of globe rupture at 36 000 J/m^2^ and 8.7 J respectively in human eyes, a significantly lower threshold than that required in pigs, at 71 000 J/m^2^ and 11.1 J respectively [57]. The thresholds were different for various projectiles (Table 2). Kumar et al had previously found iridodialysis was easier to create by repeated impacts in human eyes in vitro than in pigs, where it took about three times as many hits. In humans, it occurred after a 0.38 J impact using cylinders 6 mm in diameter with rounded tips, resulting in an area-normalised energy of 13 450 J/m^2^ [56].

**Table 2:** Parameters leading to globe rupture in humans post-mortem.

Author (year)	Object	Diameter (mm)	Mass (g)	Velocity (m/s)	Kinetic energy (J)	Normalised energy (J/m^2^)
Delori^a^ (1969) [69]	BB	4.5	0.35	71.9	0.91	56 930
Stitzel (2002) [60]	BB	4.5	0.38	85.2	1.36	85 580^b^
Stitzel (2002) [60]	Baseball	76.1	146.5	34.4	86.7	19 060^c^
Kennedy (2006) [57]	Aluminium rod	9.25	3.57	42.1	3.17	47 230
Kennedy (2006) [57]	Paintball	17.3	3.13	65.5	6.72	28 600

^a^Calculated by Kennedy; numbers for velocity and kinetic energy are different in the original paper.

^b^The next lower value was about 42 000 J/m^2^; the threshold would lie anywhere in between.

^c^The next lower value was about 14 550 J/m^2^; again, the threshold would lie in between.

Several studies have been done on porcine eyes in vitro, which are close in size to the human eye (Table 3). Weidenthal and Schepens used eyeballs with diameters around 22–27 mm, harvested from animals about six months old [68]. This appears to have become an implicit standard. An exception is a recent paper using formalin-preserved globes that investigated momentum as a potential predictive parameter for penetration of the corneoscleral shell and found thresholds depended on projectile shape [55].

**Table 3:** Parameters leading to ocular lesions in pig eyes in vitro (RD retinal detachment, LD lens dislocation).

Author (year)	Object	Diameter (mm)	Mass (g)	Velocity (m/s)	Kinetic energy (J)	Normalised energy (J/m^2^)	Damage observed
Weidenthal & Schepens (1966) [68]	BB weight drop	presumably 4.5 n/a	0.345 272	66.4 2.4	0.76 0.81	presumably 48 000 n/a	Peripheral retinal breaks or avulsions no such lesions
Scott (2000) [58]	Steel rod with rounded end	6.35	2.6/3.5 2.6/3.5 3.5	23.8/20.7 32.3/26.2 33	0.74/0.75 1.36/1.2 1.91	23 000 to 24 000 43 000 / 38 000 60 000	Lens dislocation retinal tears or avulsions no rupture
Kennedy (2006) [57]	Paintballs BBs	17.3 4.37	3.19 0.34	100 95.9	16 1.56	68 000 104 000	Globe rupture Globe rupture
Marshall (2011) [67]	Metal or plastic airsoft BBs (spheres)	6 6 6 8	0.3 0.11 0.25 0.34	101 123 99 114	1.53 0.83 1.23 2.21	54 000 29 000 43 000 44 000	Globe rupture Globe rupture Globe rupture Globe rupture
Sponsel (2011) [59]	Paintballs	ca. 17	2.1–3.2	81–97	2 3.5 9.3 10	44 000–54 000	RD, anterior LD Angle recession, posterior LD Choroid segmentation Globe rupture

### In vivo animal models

In vivo experiments have focussed mainly on retinal damage, probably due to its clinical relevance as well as to difficulty modelling it in vitro. They concentrated on natural history rather than on energy thresholds for injury. The threshold for permanent deficits in commotio retinae remains unclear experimentally as well as clinically [76, 77].

Two early studies used primates: For weight drop on the corneas of rhesus monkeys, thresholds for contusion angle deformity were almost as high as for globe rupture, unlike in humans [70]. A BB projectile produced a contrecoup lesion in owl monkeys with commotio retinae and lasting retinal pigment epithelial changes [71].

Several studies in pigs used young animals whose breed was not specified. In 20 kg store pigs, a modified air gun applied temporally with a kinetic energy of 0.76 J caused commotio retinae near the site of impact; this study found no contrecoup lesions [72]. (Projectile mass/diameter was not given but if identical to the same authors’ later study, area-normalised energy would presumably have been 48 000 J/m^2^.) In a similar setup, a missile weighing 0.38 g was fired with just over 50 m/s and 100 m/s (resulting in impact energies of 0.49 J and 1.9 J respectively) and produced peripheral retinal tears and dialyses, consistently at the higher energy level, less consistently at the lower one, and with lower thresholds when applied to the sclera than when applied to the cornea. Projectile caliber was not recorded but was presumably 4.5 mm, resulting in a presumed area-normalised energy of 31 000 J/m^2^ vs 119 000 J/m^2^ [73]. The authors noted that injuries in vivo required higher levels of kinetic energy than similar lesions produced in vitro, perhaps because traction forces required to pull the sensory retina away from the pigment epithelium are lower post-mortem.

In pigs weighing 11–27 kg, a posterior contusion injury with intraocular haemorrhage and extensive commotio retinae but without retinal breaks or dialysis could be produced by using a 0.22 caliber pistol that applied a modified lead pellet (diameter not specified) with a mass of 0.57 g, a velocity of 33 m/s and a kinetic energy of 0.32 J temporally [74]. In similar pigs impacted on the cornea, the same authors produced a contrecoup lesion with extensive commotio retinae and structural changes of the pigment epithelium using a 0.95 g lead pellet of unknown diameter with a velocity of 52 m/s, delivering a kinetic energy of 1.25 J [75].

The differences between species are not negligible; the smaller and more delicate eyes of rats are more vulnerable to injury, pig eyes less [76]. Experiments on rats in vivo confirm yet again that small, fast projectiles cause more damage than heavier, slower ones [77].

## Discussion

The threshold for vision-threatening eye injuries from projectiles is a complex issue. There is no range of energies or velocities known to be safe, neither for humans nor for other species. Permanent visual loss in one eye constitutes a severe injury under Swiss jurisdiction. The small rubber shot currently in use was intended to avoid such outcomes. Practical experience shows that it can still cause profound and lasting damage, even from the minimum distance. The lack of objective criteria that can serve as a guideline for regulators is not a problem confined to Switzerland. The same thing has happened elsewhere: In both Israel and Chile, the minimum distance assumed as “safe” for KIPs did not prevent devastating ocular trauma [21, 27].

For scattering pellets, the question of energy thresholds is probably academic, since it is hardly conceivable that rubber shot with enough energy to deter protesters would not endanger the eye. The recent review “Lethal in Disguise” states: “The results of our analysis suggest that these weapons” (multiple projectiles) “are more dangerous than single projectiles, and restrictions on their use must be one of the first steps in limiting harm from kinetic impact projectiles.” [2] The United Nations Human Rights Office states that “ammunition containing multiple KIPs is inaccurate, indiscriminate, arbitrary, and cannot be used safely” and that “this ammunition fulfills no legitimate law enforcement purpose that cannot be achieved through the use of ammunition containing single non-metallic KIPs” [96]. In any case, lawyers have recently begun to question the legal basis for the use of rubber shot in Switzerland [8, 97]. Possible alternatives are beyond the scope of this review but include a shift towards dialogue and de-escalation, tear gas, water cannons and pepper spray, all of which are at the disposal of the Swiss police. Tasers are under evaluation.

Given the known risks of small, fast projectiles, the suggestion of accidental injury by stones or fisticuffs, or even self-harm, is not supported by the available evidence. The extensive literature on boxing and contact sports describes hardly any globe ruptures but numerous orbital fractures [98]. Stones hurled by protesters are usually bigger than a rubber pellet and one would also expect broken bones. Severe self-inflicted eye injuries have been described only in profoundly disturbed mental states, usually florid psychosis [99].

Others are grappling with similar questions. After rupture of an eye was acknowledged as severe bodily injury by Canadian courts, a literature emerged on minimum velocities of various projectiles necessary to penetrate porcine eyes in vitro, without mentioning the inter-species differences [55, 66, 67]. A recent paper states: “The Canadian Judicial System has accepted (…) the conclusion that the penetration of an eye is the *minimum* requirement to constitute serious bodily injury” [55] (emphasis added). Such a definition would differ from the one in Switzerland, where the relevant requirement is visual loss, regardless of the anatomical basis.

In other democracies that use KIPs, for example France and the United States, ophthalmologists and other physicians published case series in medical journals soon after observing significant injuries [26, 28]. They also made clear statements about the risks of kinetic impact projectiles, not only in medical journals [100, 101] but also in public [102–105]. In consequence, eye injuries from KIPs have become less frequent in both countries [personal communications from Dr. Bahram Bodaghi, Paris, France, and Dr. Prem Subramanian, Colorado, USA]. There are now plans for an ophthalmological case series in Switzerland as well.

Thresholds for ocular damage from projectiles are relevant for various situations, including eye injuries from toys. Such accidents are on the rise due to the introduction of ever more varied and sophisticated toy guns [93]. Reporting kinetic and area-normalised energies in these cases would provide insight into potential differences in vulnerability between the eyes of children and adults.

## Conclusion

Policymakers need to be aware of the risks of less-lethal weapons so that they can balance their use with other options of crowd control and set appropriate limits. Neither judges [106] nor specialists in forensic medicine [107, 108] can be expected to understand the details of ocular vulnerability to injury. It is therefore critical for the ophthalmological profession to be involved in the evaluation of both such weapons and the eye injuries they produce. Underreporting the risks of kinetic impact projectiles may contribute to their prolonged and increasingly liberal use. Closer collaboration between ballistics and forensics specialists and ophthalmologists would provide a unique opportunity to correlate the physical parameters discussed above with the clinical sequels. The results would be of obvious interest for all kinds of projectiles including toys.

## Summary

### What was known before

- Eye injuries by kinetic impact projectiles (KIPs) have been reported in several countries, including Switzerland.
- Most eye injuries by KIPs occur from multiple projectiles.

### What this study adds

- Switzerland has a longstanding, ongoing series of eye injuries by rubber scattershot.
- Underreporting the risks of crowd-control weapons may lead to their prolonged and increasingly liberal use.
- An interdisciplinary approach including ballistics, forensics and ophthalmology could help to elucidate energy thresholds for visual loss.
