# Correction: Risks and regulation of rubber scattershot in Switzerland: a narrative review

**DOI:** 10.1038/s41433-024-03291-y

**Published:** 2024-08-23

**Authors:** Anna Fierz

**Affiliations:** Ophthalmologist in private practice, Kalkbreitestr. 8, 8003 Zürich, Switzerland

**Keywords:** Risk factors, Physiology

Correction to: *Eye* 10.1038/s41433-024-03215-w, published online 08 July 2024

In Table 1, the number of KIPs for Cougar-56 (Alsetex) has been corrected from ‘29’ to ‘20’. The reference for this number has also been corrected to “Schöni B. (How the police shoot at demonstrators.) German. Republik.ch, 23.7.24, https://republik.ch/2024/07/23/wie-die-polizei-auf-demonstrierende-schiesst”. The original article has been corrected.

Table 1 in the original article:

**Table 1:** Kinetic impact projectiles (KIPs) used by Swiss law enforcement agencies.

Type	Size (mm)	Weight (g)	Muzzle velocity (m/s)	Minimum distance (m)	Kinetic energy at MD (J)	Area-normalised energy at MD (J/m^2^)	Number of KIPs	Dispersion area at distance given (m)
MZW rubber prisms (Saltech)	Length 26-27, hexagon sides 10 [41, own measurement]	10 [38]	70 [38]	20	10.2 [38]	39 000 [38]	35	about 4×4 at MD [8]
GL-06 rubber shot (B&T)	Diameter 15 [41]	2.6 [41]	88 [41]	5 [42]	about 8 [41]	about 45 000	28 [41]	about 2.7×3.3 (at 20 m) [41]
GL-06 “Rubber Shot Hexagonal” (Saltech)	Length 18, hexagon sides smaller than in MZW prisms (Fig. 1)	8.7 [42]	?	10 [42]	?	?	28 [42]	allegedly smaller than for MZW rubber prisms [43]
GL-06 SIR (B&T)	40×99 [40]	77 [40]	85 [40]	5 [42]	about 100-110 [50]	about 83 000	1	-
Cougar-56 (Alsetex)	?	?	?	15 [44]	?	?	29 [44]	allegedly about 1×1 (at MD) [44]

*MZW* Mehrzweckwerfer, *MD* minimum distance, *SIR* “Safe Impact Round”.

Corrected Table 1:

**Table 1:** Kinetic impact projectiles (KIPs) used by Swiss law enforcement agencies.

Type	Size (mm)	Weight (g)	Muzzle velocity (m/s)	Minimum distance (m)	Kinetic energy at MD (J)	Area-normalised energy at MD (J/m^2^)	Number of KIPs	Dispersion area at distance given (m)
MZW rubber prisms (Saltech)	Length 26–27, hexagon sides 10 [41, own measurement]	10 [38]	70 [38]	20	10.2 [38]	39,000 [38]	35	About 4 × 4 at MD [8]
GL-06 rubber shot (B&T)	Diameter 15 [41]	2.6 [41]	88 [41]	5 [42]	About 8 [41]	About 45,000	28 [41]	About 2.7 × 3.3 (at 20 m) [41]
GL-06 “Rubber Shot Hexagonal” (Saltech)	Length 18, hexagon sides smaller than in MZW prisms (Fig. 1)	8.7 [42]	?	10 [42]	?	?	28 [42]	Allegedly smaller than for MZW rubber prisms [43]
GL-06 SIR (B&T)	40 × 99 [40]	77 [40]	85 [40]	5 [42]	About 100–110 [50]	About 83,000	1	–
Cougar-56 (Alsetex)	?	?	?	15 [44]	?	?	20 [109]	Allegedly about 1 × 1 (at MD) [44]

*MZW* Mehrzweckwerfer, *MD* minimum distance, *SIR* “Safe Impact Round”.

